# Candida auris Forms High-Burden Biofilms in Skin Niche Conditions and on Porcine Skin

**DOI:** 10.1128/mSphere.00910-19

**Published:** 2020-01-15

**Authors:** Mark V. Horton, Chad J. Johnson, John F. Kernien, Tarika D. Patel, Brandon C. Lam, J. Z. Alex Cheong, Jennifer J. Meudt, Dhanansayan Shanmuganayagam, Lindsay R. Kalan, Jeniel E. Nett

**Affiliations:** a Department of Medicine, University of Wisconsin, Madison, Wisconsin, USA; b Department of Medical Microbiology and Immunology, University of Wisconsin, Madison, Wisconsin, USA; c Department of Animal Science, University of Wisconsin, Madison, Wisconsin, USA; Carnegie Mellon University

**Keywords:** *Candida auris*, biofilm, pathogenicity, skin, porcine, sweat, transmission

## Abstract

Emerging pathogen Candida auris causes nosocomial outbreaks of life-threatening invasive candidiasis. It is unclear how this species colonizes skin and spreads in health care facilities. Here, we analyzed C. auris growth in synthetic sweat medium designed to mimic axillary skin conditions. We show that C. auris demonstrates a high capacity for biofilm formation in this milieu, well beyond that observed for the most commonly isolated *Candida* sp., Candida albicans. The C. auris biofilms persist in environmental conditions expected in the hospital setting. To model C. auris skin colonization, we designed an *ex vivo* porcine skin model. We show that C. auris proliferates on porcine skin in multilayer biofilms. This capacity to thrive in skin niche conditions helps explain the propensity of C. auris to colonize skin, persist on medical devices, and rapidly spread in hospitals. These studies provide clinically relevant tools to further characterize this important growth modality.

**IMPORTANCE** The emerging fungal pathogen Candida auris causes invasive infections and is spreading in hospitals worldwide. Why this species exhibits the capacity to transfer efficiently among patients is unknown. Our findings reveal that C. auris forms high-burden biofilms in conditions mimicking sweat on the skin surface. These adherent biofilm communities persist in environmental conditions expected in the hospital setting. Using a pig skin model, we show that C. auris also forms high-burden biofilm structures on the skin surface. Identification of this mode of growth sheds light on how this recently described pathogen persists in hospital settings and spreads among patients.

## Abstract (precis)

The emerging fungal pathogen Candida auris causes invasive infections and is spreading in hospitals worldwide. Why this species exhibits the capacity to transfer efficiently among patients is unknown. Our findings reveal that C. auris forms high-burden biofilms in conditions mimicking sweat on the skin surface. These adherent biofilm communities persist in environmental conditions expected in the hospital setting. Using a pig skin model, we show that C. auris also forms high-burden biofilm structures on the skin surface. Identification of this mode of growth sheds light on how this recently described pathogen persists in hospital settings and spreads among patients.

## INTRODUCTION

For patients with invasive Candida auris infections, mortality rates can be exceedingly high, approaching 60% (1). Due to the severity of the infections and ongoing nosocomial outbreaks, C. auris has emerged as the first fungal pathogen to be designated a global public health threat (2). In the current report of antibiotic resistance threats in the United States, the Centers for Disease Control and Prevention lists C. auris as the highest-level threat. In health care settings, C. auris efficiently colonizes the skin, persists on hospital surfaces, and rapidly spreads among patients (1, 3, 4). As other *Candida* species typically colonize the gastrointestinal tract, the apparent propensity for C. auris to persist on the skin surface is a distinct trait, likely contributing to its efficient nosocomial transmission. However, despite the growing concern for C. auris infection within health care facilities, little is known regarding the mechanism of C. auris skin colonization.

Similarly to other *Candida* species, C. auris infections typically occur in patients with indwelling medical devices, such as vascular catheters, G tubes, and endotracheal tubes (1, 5). On these artificial surfaces, *Candida* spp. produce biofilms, adherent microbial communities encased within a protective extracellular matrix (6, 7). While prior studies have demonstrated biofilm formation for C. auris, the observed structures are not nearly as dense as those formed by Candida albicans, the most prevalent *Candida* sp. (8–12). Due to the predilection of C. auris for skin, we questioned if C. auris may exhibit an enhanced capacity to proliferate in the cutaneous environment, whereby it could gain access to the bloodstream via the insertion and continued presence of vascular catheters. Here, we describe the remarkable ability of C. auris to form biofilms in conditions of the skin environment, mimicked experimentally using synthetic human sweat medium and an *ex vivo* porcine skin model.

## RESULTS

### C. auris forms high-burden biofilms in skin milieu.

**FIG 1:**
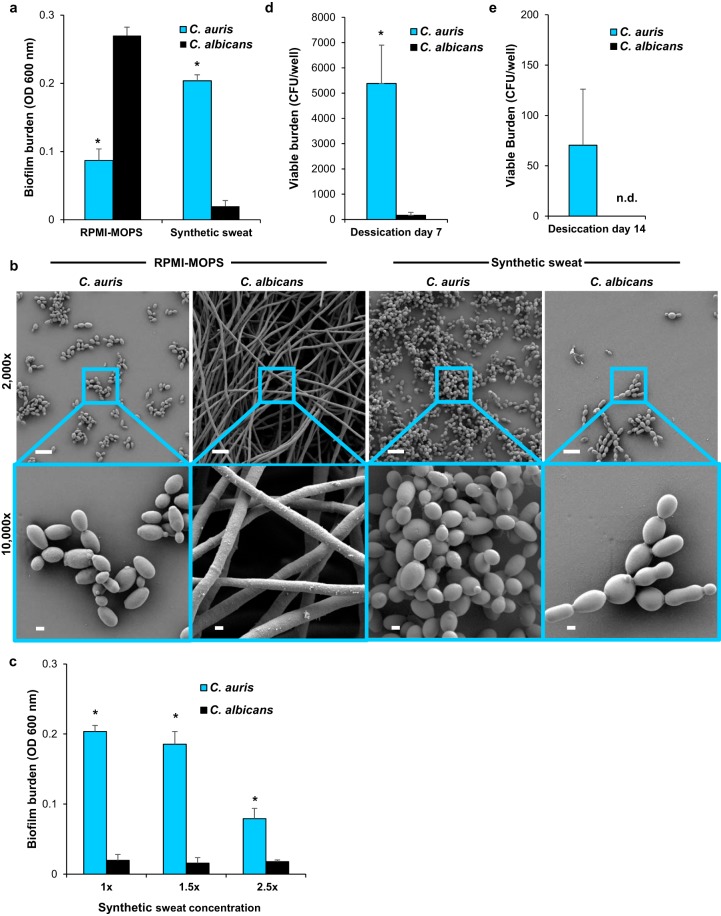
C. auris forms high-burden biofilms in synthetic sweat medium. (a) *Candida* biofilms were grown in RPMI-MOPS or synthetic sweat medium for 24 h, and biofilm burden was measured by absorbance at 600 nm. C. auris biofilm density was compared to that of C. albicans in each media by Student's *t* test, *, *P* < 0.05, standard errors of the means shown, *n* = 3. (b) *Candida* biofilms were grown on coverslips (24 h) and imaged with scanning electron microscopy. Bars, 10 μm and 1 μm for ×2,000 and ×10,000 magnification images, respectively. (c) *Candida* biofilms were grown in various concentrations of synthetic sweat medium for 24 h, *, *P* < 0.05 by Student's *t* test at each concentration, standard errors of the means shown, *n* = 3. (d and e) *Candida* biofilms were subjected to 24 h of desiccation, and viable burdens were assessed by plating of serial dilutions of disrupted biofilms after 7 or 14 days, *, *P* < 0.05 by Student's *t* test, standard errors of the means shown, *n* = 6; n.d., not detected.

To assess the capacity of C. auris to proliferate in skin niche conditions, we produced synthetic sweat medium, designed to mimic human axillary sweat, and examined biofilm formation. After 24 h of incubation, C. auris produced dense biofilms with 10-fold greater burden than the biofilms formed by C. albicans (Fig. 1a) (*P* < 0.05). This is quite striking, as under typical laboratory conditions, C. albicans exhibits a capacity for biofilm formation well beyond that observed for most other *Candida* spp. (13). For example, in RPMI-MOPS (morpholinepropanesulfonic acid), the biomass of biofilms formed by C. albicans was 3-fold greater than for C. auris (Fig. 1a) (*P* < 0.05), consistent with prior studies demonstrating lower biofilm burdens for this species (8, 9).

To evaluate the biofilm architecture and cellular morphology of C. auris growing in skin niche conditions, we grew biofilms on coverslips and imaged them by scanning electron microscopy. Consistent with quantitative biofilm formation assays, C. auris growing in synthetic sweat medium replicated that on the coverslip surface, proliferating as a multilayer biofilm, composed entirely of yeast cells (Fig. 1b). In contrast, C. albicans formed a very rudimentary biofilm, composed of yeast with rare pseudohyphae or hyphae. Biofilm structures and burdens were vastly different for biofilms grown in RPMI-MOPS. In this tissue culture medium, C. albicans formed dense biofilms composed primarily of hyphae, while biofilms formed by C. auris appeared as a monolayer of yeast. These findings show that C. auris exhibits a heightened capacity to form biofilms in the milieu of the skin surface.

Circulating C. auris strains vary genetically and cluster into at least 4 distinct clades (1). The C. auris strain (B11203) selected for our initial experiments had been isolated from a patient in India and phylogenetically placed in the South Asian or India/Pakistan clade (1). Given the genetic diversity of strains, we questioned if the capacity for robust biofilm formation in skin niche conditions may represent a shared trait among C. auris strains. To address this question, we obtained a strain set from the Centers for Disease Control (see Table S1 in the supplemental material) which encompassed at least one isolate from each clade, and we examined biofilm formation (1). Biofilm growth by the majority of the strains paralleled that observed for C. auris B11203 (listed as strain 5), with burdens reaching approximately 10-fold that of C. albicans (see Fig. S1). We next considered if the differences in biofilm formation may be a consequence of general medium-specific growth differences. Indeed, the majority of C. auris strains appeared to have a slight growth advantage over C. albicans in the sweat medium under nonbiofilm conditions (see Fig. S2). However, these relatively small differences did not account for the 10-fold greater biofilm formation observed for C. auris (Fig. 1a and S1). The findings show that robust biofilm formation in skin niche conditions is a common characteristic for C. auris. As C. auris B11203 appeared representative of the C. auris isolates, we continued to utilize this strain for subsequent experiments.

10.1128/mSphere.00910-19.1 FIG S1 Diverse strains of C. auris form biofilms in synthetic sweat. *Candida* biofilms were grown in synthetic sweat medium, and burdens were measuring by absorbance at 600 nm. C. auris biofilm burdens were analyzed using one-way analysis of variance (ANOVA) with Holm-Sidak posttest comparison to control (C. albicans). *, *P* < 0.05, standard deviations shown, *n* = 4. Download FIG S1, PDF file, 0.1 MB. Copyright © 2020 Horton et al. 2020 Horton et al. This content is distributed under the terms of the Creative Commons Attribution 4.0 International license.

10.1128/mSphere.00910-19.2 FIG S2 Planktonic growth of C. auris in synthetic sweat. Strains were grown in synthetic sweat medium at 37°C with shaking for 16 h. C. auris growth was analyzed using one-way ANOVA with Holm-Sidak posttest comparison to control (C. albicans); no statistical differences, standard deviations shown, *n* = 3. Download FIG S2, PDF file, 0.1 MB. Copyright © 2020 Horton et al. 2020 Horton et al. This content is distributed under the terms of the Creative Commons Attribution 4.0 International license.

10.1128/mSphere.00910-19.3 TABLE S1 C. auris strains used in this study. Download Table S1, DOCX file, 0.1 MB. Copyright © 2020 Horton et al. 2020 Horton et al. This content is distributed under the terms of the Creative Commons Attribution 4.0 International license.

### C. auris biofilms formed in skin niche conditions resist desiccation.

Next, we questioned if the formation of biofilm by C. auris in the skin milieu environment may serve as a mechanism to resist desiccation. We reasoned that desiccation might occur in two environments, either on the skin surface as sweat evaporates or in the environment where sweat and desquamated skin dry on surfaces. As sweat evaporates, the concentration of salt and other components increases. To mimic this process on the skin surface, we concentrated synthetic sweat medium and examined the influence on biofilm formation. In 1.5×-concentrated synthetic sweat, C. auris formed high-burden biofilms, 10-fold greater than C. albicans, mirroring the findings observed for the nonconcentrated sweat (Fig. 1c). Even in 2.5×-concentrated sweat, C. auris exhibited the capacity to proliferate as a biofilm. The burdens of these biofilms were below those formed in the less-concentrated sweat but were still 4-fold greater than the biofilms formed by C. albicans.

To model environmental drying of sweat and desquamated skin, we produced biofilms in synthetic sweat medium and subjected the biofilms to 24 h of desiccation in a laminar flow hood. At 7 days postdesiccation, we found C. auris to persist at a burden approximately 30-fold greater than C. albicans (Fig. 1d). At 14 days postdesiccation, C. auris grew readily from biofilms, while C. albicans biofilms were not viable (Fig. 1e). Taken together, these results illustrate that biofilms formed by C. auris in conditions of the skin microenvironment persist despite conditions of both sweat evaporation and environmental desiccation.

### C. auris forms high-burden biofilms in skin niche conditions on porcine skin.

**FIG 2:**
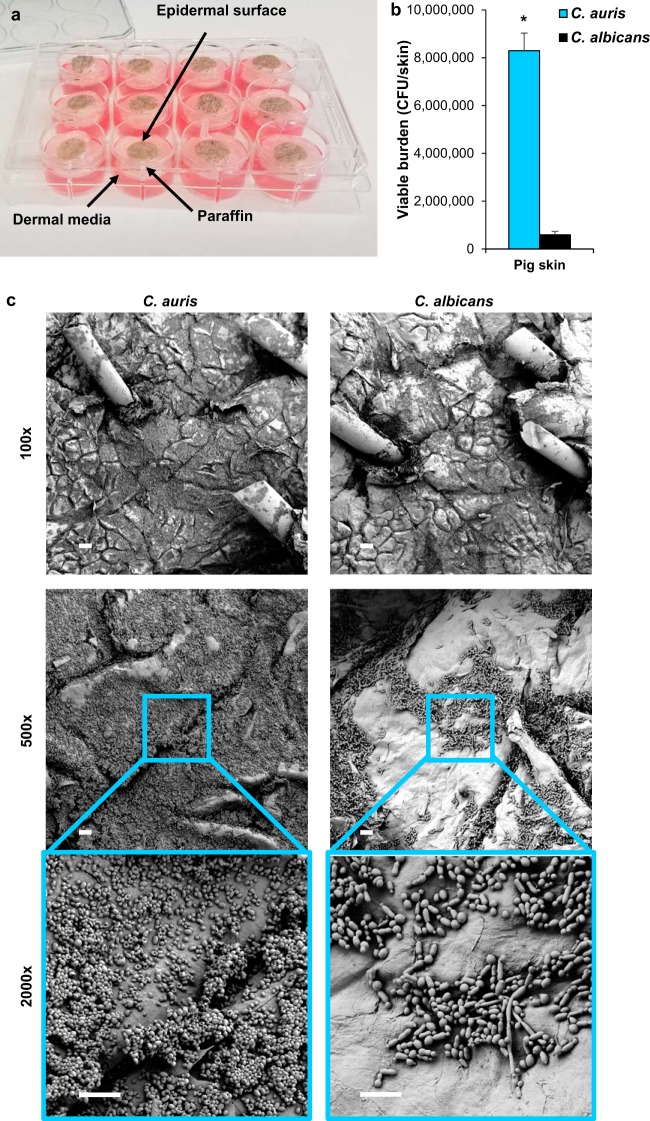
C. auris effectively colonizes porcine skin. (a) Porcine skin samples were placed in DMEM supplemented with 10% FBS and set in paraffin to separate the epidermal surface from the liquid medium. *Candida* cells suspended in synthetic sweat medium were inoculated on the epidermal surface. (b) *Candida* cultures were suspended in synthetic sweat medium inoculated on the surface of porcine skin samples. Following 24 h of incubation, *Candida* growth on the hair and skin surface was enumerated via CFU counts. *, *P* < 0.05 by Student's *t* test, standard errors of the means shown, *n* = 4. (c) *Candida* biofilms were grown on porcine skin samples and imaged with scanning electron microscopy. Bars, 100 μm, 20 μm, and 10 μm for ×100, ×500, and ×2,000 magnification, respectively.

To simulate C. auris growth in the skin niche, we developed an *ex vivo* porcine skin model employing the Wisconsin Miniature Swine (14). Pig skin was selected based on the many characteristics it shares with human skin, including similarities in the thickness of skin layers, the types and distribution of skin cells, and the mechanisms of skin repair (15–18). As conventional pigs have been bred for increased size and muscle mass, miniature varieties more closely mimic human body composition (14, 15). Full-thickness skin samples were harvested from excised skin and placed in semisolid medium to supply nutrients to the dermis (Fig. 2a). The addition of paraffin to the medium-epidermal interface provided a barrier between the dermal medium and the skin surface, allowing these compartments to remain separated and limiting *Candida* growth to the skin surface. Following inoculation of the epidermis with *Candida* suspended in synthetic sweat medium, we allowed the samples to incubate for 24 h, at which time the sweat medium had evaporated. Consistent with our *in vitro* observations, C. auris exhibited enhanced growth compared to that of C. albicans in this environment, with C. auris propagating to a burden nearly 15-fold greater than C. albicans and replicating nearly 100-fold above the initial inoculum (Fig. 2b) (*P* < 0.05).

We next utilized scanning electron microscopy to assess the distribution of C. auris biomass, the architecture of skin-associated biofilms, and the interaction of *Candida* with the epidermal surface. Following inoculation of C. auris, imaging of the porcine skin at low magnification revealed diffuse fungal growth over the epidermal surface (Fig. 2c). Higher magnification imaging showed the formation of multilayer biofilms composed of yeast cells. In contrast, imaging of porcine skins inoculated with C. albicans showed little fungal growth. Multilayer aggregates were not observed. Examination of the fungal-epithelial interface for both species revealed intact skin without evidence of tissue invasion. This lack of invasion supports the use of porcine skin to model human skin colonization, as this process does not involve tissue destruction by fungi. Furthermore, the results reveal that C. auris exhibits an enhanced propensity to proliferate on porcine skin.

## DISCUSSION

The results of these studies shed light on the capacity of C. auris to proliferate and persist on the skin surface and in environmental conditions involving skin components. Our findings demonstrate that growth in synthetic sweat medium and on porcine skin allows C. auris to form dense biofilms that flourish in conditions of evaporation and resist desiccation. These observations help to explain this pathogen’s propensity for colonizing skin, persisting on hospital surfaces, and spreading efficiently in the hospital setting (4, 19). As an example, a C. auris outbreak in the United Kingdom was linked to the use of reusable axillary temperature probes (20). Given the axillary skin environment in this setting, we propose that the outbreak likely involved robust biofilm growth similar to that observed in the present study. We also postulate that other reusable equipment, such as blood pressure cuffs and intravenous infusion pumps, can harbor resilient C. auris biofilms. These items are also in frequent contact with skin and often shared among patients.

Our finding that C. auris resists desiccation during biofilm formation may not be surprising, as a prior study has shown this species to survive dry conditions for up to 2 weeks (21). Little is known about the mechanism(s) utilized by C. auris to resist desiccation. However, studies by Day and colleagues characterized the responses of *Candida* to a variety of stresses, including high salinity, and showed that C. auris exhibited a unique stress resistance profile compared to those of other *Candida* spp. (22). Despite the different profiles, these responses involved the evolutionarily conserved Hog1 stress-activated protein kinase (SAPK). It is intriguing to speculate that biofilm formation and resistance to desiccation may be intertwined with this pathway. In addition, the karyotypic plasticity displayed by C. auris in response to stressors may contribute to this organism’s ability to adapt and persist in the skin niche (23). Further understanding of these stress tolerance pathways may lead to identifying new approaches to eradicate C. auris from the environment, to prevent person-to-person spread, or to pharmaceutically target C. auris.

What is striking about C. auris is that individual strains appear to have arisen independently in remote geographic locations (1). While an environmental niche for C. auris has not been described, one hypothesis is that increasing temperatures due to climate change promoted the emergence of this species that could then thrive in the high temperatures of mammalian and avian hosts (24). Because biofilm formation is a mechanism for adherence and persistence in various environments, insight into factors governing C. auris robust biofilms may also help elucidate environmental factors that have contributed to the emergence of this organism.

The present study provides tools to further dissect C. auris growth and biofilm formation in the skin environment. In light of the similarities to human skin, porcine skin serves as an excellent model for studies of wound healing, infection, and skin colonization (15–18, 25). Growth of C. auris in this *ex vivo* model mimics what is observed clinically, an enhanced capacity for skin colonization. Understanding the interactions of C. auris with skin and the pathways involved in biofilm formation may provide insight into new strategies to control the spread of this emerging pathogen.

## MATERIALS AND METHODS

### Organisms and inoculum.

Studies included C. albicans SC5314 and C. auris strains provided by the Centers for Disease Control and Prevention (see Table S1 in the supplemental material) (1). Strains were maintained on yeast extract-peptone-dextrose (YPD) plates and propagated in YPD broth overnight in an orbital shaker at 30°C. Overnight cultures were diluted 1:1,000 in Dulbecco’s phosphate-buffered saline (DPBS), vortexed thoroughly, counted with a hemocytometer, and adjusted to 10^6^ cells/ml in RPMI-MOPS or synthetic sweat medium.

### Synthetic sweat medium.

Artificial human axillary sweat medium was adapted from that described by Callewaert et al. (26) with several modifications (Table S2). In place of fatty acids hydrolyzed from human abdominal subcutaneous fat, we included a mix of the three most abundance fatty acids found in human sweat (lauric, myristic, and palmitic fatty acids) (26, 27). In light of this, lower concentrations of fatty acids were added to ensure solubility. Cholesterol and lactic acid concentrations were selected based on prior measurements for sweat (28, 29). Medium was made at a 2.5× concentration, warmed to 60°C with stirring, and filter sterilized. Medium was stored at room temperature and diluted to 1× concentration with sterile water.

10.1128/mSphere.00910-19.4 TABLE S2 Synthetic sweat medium composition. Download Table S2, DOCX file, 0.1 MB. Copyright © 2020 Horton et al. 2020 Horton et al. This content is distributed under the terms of the Creative Commons Attribution 4.0 International license.

### Biofilm and planktonic growth.

For biofilm assays, 200 μl was added to flat-bottom 96-well microtiter plates (30). Following a 24 h of incubation at 37°C, biofilms were rinsed gently with DPBS, and 200 μl of DPBS was applied. The biofilm burdens were then measured by optical density at 600 nm (OD_600_) values obtained using a microplate reader (Synergy H1; Bio-Tek Instruments) (31). For planktonic growth curves, 200 μl was similarly seeded in flat-bottom 96-well microtiter plates. Plates were then placed in the microplate reader with shaking at 37°C, and OD_600_ values were obtained.

### Desiccation assay.

Biofilms were grown in flat-bottom 96-well microtiter plates for 24 h, as described above. After removing the medium and nonadherent cells, plates were desiccated in a laminar flow hood with lids removed for 24 h and subsequently incubated at room temperature. After 7 or 14 days, biofilms were harvested, vortexed, and plated on YPD agar for determination of viable burden.

### Porcine skin model.

The collection of porcine skin samples was conducted under protocols approved by the University of Wisconsin—Madison Institutional Animal Care and Use Committee in accordance with published National Institutes of Health (NIH) and United States Department of Agriculture (USDA) guidelines. Skin from euthanized animals was excised, washed alternately with DPBS and 70% ethanol (ETOH) until clean, and then shaved. Using a biopsy punch, 12-mm full-thickness skin samples were extracted and placed into the wells of 12-well plates, each containing 3 ml Dulbecco’s modified Eagle medium (DMEM) supplemented 10% fetal bovine serum (FBS) (10%), penicillin (1,000 U/ml), and streptomycin (1 mg/ml). Samples were incubated at 37°C with 5% CO_2_ for 18 h with one change of medium after 6 h. Tissues were then rinsed in DPBS, dried thoroughly, and placed on semisolid medium (6:4 ratio of 1% agarose in DPBS and DMEM with 10% FBS). To construct a barrier to separate the subdural medium (DMEM) and epidermal surface, 800 μl paraffin wax was applied to the inner surface of the DMEM at the epidermal interface. C. auris suspended in synthetic sweat medium (10^7^ cells/ml) was applied to the skin surface (10 μl). Following 24 h of incubation without a humidity source at 37°C with 5% CO_2_, skin samples were processed for scanning electron microscopy (described below) or vortexed in DPBS and plated for assessment of viable burden.

### Scanning electron microscopy.

*Candida* biofilms were examined by scanning electron microscopy as previously described (32, 33). Briefly, 13-mm Thermanox coverslips were coated with 4 μg/ml fibrinogen in DPBS for 1 h at 37°C, rinsed twice with water, and dried for 2 h. Cultures of *Candida* (40 μl at 1.5 × 10^7^ cells/ml cultures in RPMI-MOPS or synthetic sweat medium) were added for 30 min, and then nonadherent cells were removed. Following replacement of the medium, coverslips were incubated at 37°C for 24 h, washed, and fixed overnight (4% formaldehyde, 1% glutaraldehyde, in DPBS). Samples were next washed with DPBS, treated with 1% osmium tetroxide, and then washed with DPBS. Samples were dehydrated with multiple ethanol washes, subjected to critical point drying, mounted on aluminum stubs, sputter coated with platinum, and imaged with a scanning electron microscope (LEO 1530) at 3 kV. Pig skin samples were similarly processed and mounted on aluminum stubs with carbon paint, and silver paint was applied around the perimeter for improved conductivity.
