# Supplementary material for: Candida auris Forms High-Burden Biofilms in Skin Niche Conditions and on Porcine Skin
Source: mSphere. 2020 Jan 15;5(1):e00910-19. doi: 10.1128/mSphere.00910-19 (PMC6977180; doi:10.1128/mSphere.00910-19)
Supplement: FIG S1 [file mSphere.00910-19-sf001.pdf]

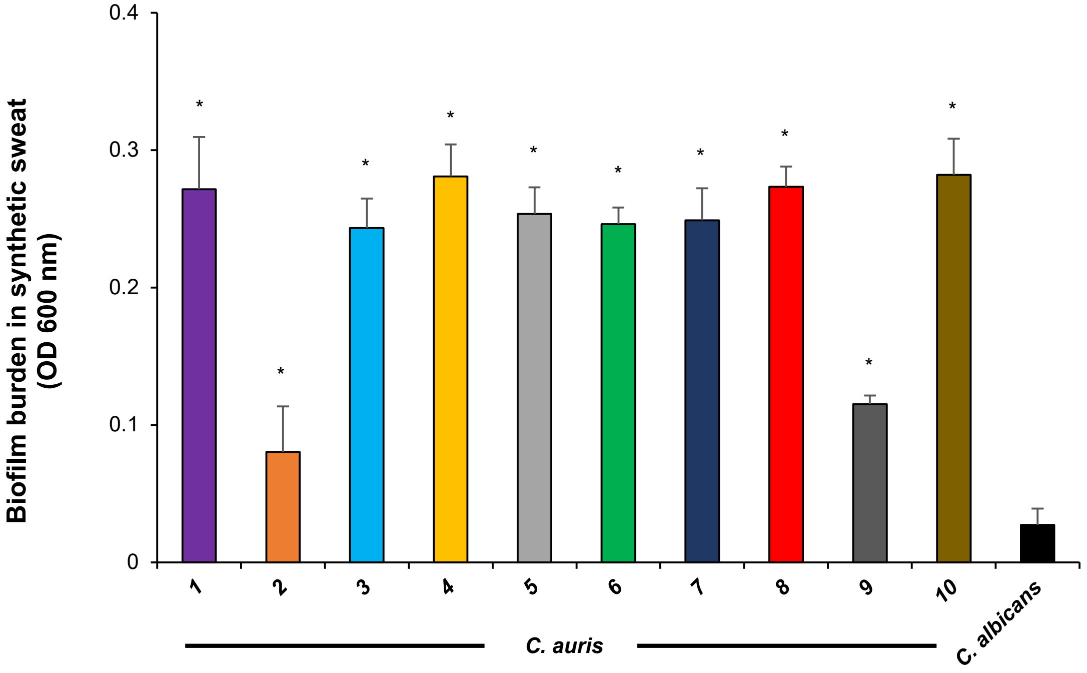

Fig. S1. **Diverse strains of *C. auris* form biofilms in synthetic sweat**. *Candida* biofilms were grown in synthetic sweat media and burdens were measuring by absorbance at 600nm. *C. auris* biofilm burdens were analyzed using one-way ANOVA with Holm-Sidak post-test comparison to control (*C. albicans*), *P<0.05, standard deviation shown, n=4.
