# Supplementary material for: Candida auris Forms High-Burden Biofilms in Skin Niche Conditions and on Porcine Skin
Source: mSphere. 2020 Jan 15;5(1):e00910-19. doi: 10.1128/mSphere.00910-19 (PMC6977180; doi:10.1128/mSphere.00910-19)
Supplement: FIG S2 [file mSphere.00910-19-sf002.pdf]

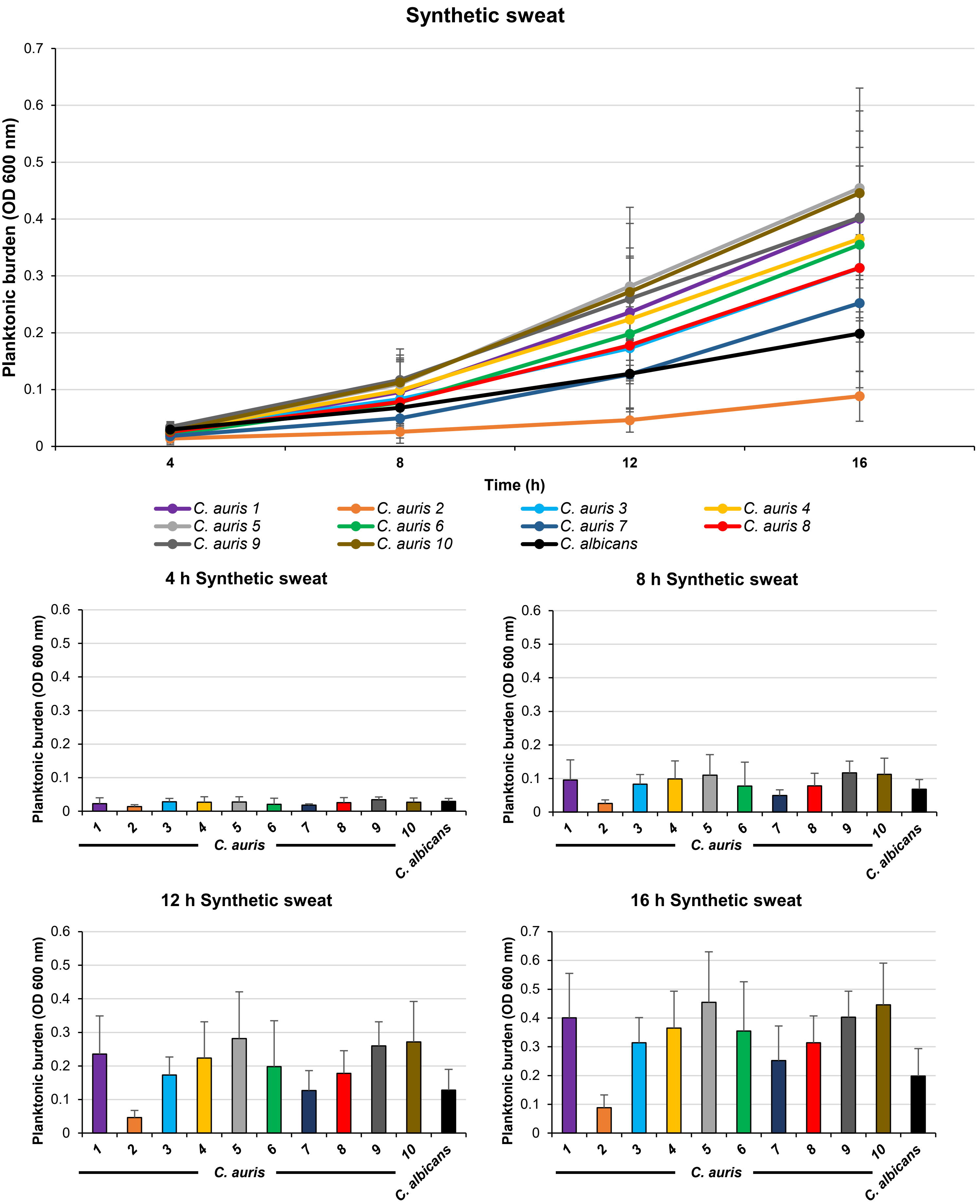

Fig. S2. Planktonic growth of *C. auris* in synthetic sweat. Strains were grown in synthetic sweat media at 37°C with shaking for 16 h. *C. auris* growth was analyzed using one-way ANOVA with Holm-Sidak post-test comparison to control (*C. albicans*), no statistical differences, standard deviation shown, n=3.
