# Supplementary material for: Candida auris Forms High-Burden Biofilms in Skin Niche Conditions and on Porcine Skin
Source: mSphere. 2020 Jan 15;5(1):e00910-19. doi: 10.1128/mSphere.00910-19 (PMC6977180; doi:10.1128/mSphere.00910-19)
Supplement: TABLE S1 [file mSphere.00910-19-st001.docx]

**Supplementary Table 1: *C. auris* strains used in this study**

| **Listed number** | ***C. auris* strain** | **Country of origin** |
| --- | --- | --- |
| 1 | B11804 | Columbia |
| 2 | B11220 | Japan |
| 3 | B11221 | South Africa |
| 4 | B11801 | Columbia |
| 5 | B11203 | India |
| 6 | B11219 | India |
| 7 | B11211 | India |
| 8 | B11104 | Pakistan |
| 9 | B11799 | Columbia |
| 10 | B11785 | Columbia |
