# Supplementary material for: Candida auris Forms High-Burden Biofilms in Skin Niche Conditions and on Porcine Skin
Source: mSphere. 2020 Jan 15;5(1):e00910-19. doi: 10.1128/mSphere.00910-19 (PMC6977180; doi:10.1128/mSphere.00910-19)
Supplement: TABLE S2 [file mSphere.00910-19-st002.docx]

**Supplementary Table 2: Synthetic sweat media composition**

| **Compound** | **Amount (% (w/v))** |
| --- | --- |
| Sodium Chloride | 2.923 |
| Lactic acid (88%) | 0.1441 |
| Urea | 1.201 |
| Glycine | 0.125 |
| L-Leucine | 0.026 |
| L-Cysteine | 0.003 |
| L-Serine | 0.303 |
| L-Alanine | 0.081 |
| L-Arginine | 0.001 |
| L-Histidine | 0.043 |
| L-Valine | 0.032 |
| L-Isoleucine | 0.020 |
| L-Lysine | 0.021 |
| L-Phenylalanine | 0.021 |
| L-Tyrosine | 0.034 |
| L-Glutamine | 0.003 |
| L-Aspartic Acid | 0.055 |
| D-Glucose | 0.180 |
| Creatinine | 0.004 |
| Sodium Pyruvate | 0.055 |
| Potassium H Carbonate | 0.120 |
| NaH_2_PO_4_ | 0.002 |
| Calcium Sulfate | 0.057 |
| Squalene | 0.002 |
| Cholesterol | .0008 |
| Fatty Acids  25% Lauric Acid  25% Palmitic Acid  50% Myristic acid | .0164 |
